# Observation and analysis of structural changes in fused silica by continuous irradiation with femtosecond laser light having an energy density below the laser-induced damage threshold

**DOI:** 10.3762/bjnano.5.146

**Published:** 2014-08-21

**Authors:** Wataru Nomura, Tadashi Kawazoe, Takashi Yatsui, Makoto Naruse, Motoichi Ohtsu

**Affiliations:** 1 School of Engineering, The University of Tokyo, and The Nanophotonics Research Center, The University of Tokyo, 2-11-16 Yayoi, Bunkyo-ku, Tokyo, 113-8656 Japan,; 2 National Institute of Information and Communications Technology, 4-2-1 Nukui-kita, Koganei, Tokyo 184-8795, Japan

**Keywords:** compositional change in molecular, femtosecond laser, fused silica, laser-induced damage, laser-induced degradation

## Abstract

The laser-induced damage threshold (LIDT) is widely used as an index for evaluating an optical component’s resistance to laser light. However, a degradation in the performance of an optical component is also caused by continuous irradiation with laser light having an energy density below the LIDT. Therefore, here we focused on the degradation in performance of an optical component caused by continuous irradiation with femtosecond laser light having a low energy density, i.e., laser-induced degradation. We performed an in situ observation and analysis of an increase in scattering light intensity in fused silica substrates. In experiments conducted using a pulsed laser with a wavelength of 800 nm, a pulse width of 160 fs and pulse repetition rate of 1 kHz, we found that the scattered light intensity increased starting from a specific accumulated fluence, namely, that the laser-induced degradation had a threshold. We evaluated the threshold fluence *F*_t_ as 6.27 J/cm^2^ and 9.21 J/cm^2^ for the fused silica substrates with surface roughnesses of 0.20 nm and 0.13 nm in *R*_a_ value, respectively, showing that the threshold decreased as the surface roughness increased. In addition, we found that the reflected light spectrum changed as degradation proceeded. We analyzed the details of the degradation by measuring instantaneous reflectance changes with a pump–probe method; we observed an increase in the generation probability of photogenerated carriers in a degraded silica substrate and a damaged silica substrate and observed a Raman signal originating from a specific molecular structure of silica. From these findings, we concluded that compositional changes in the molecular structure occurred during degradation due to femtosecond laser irradiation having an energy density below the LIDT.

## Introduction

Since the invention of the laser, it has been widely known that damages occur at the surface or interior of optical components when irradiated with laser light having a high energy density. A lot of research has been conducted in the area of laser-induced damage. For example: mechanism for picosecond [1–2] and femtosecond (fs) laser [3–4], effect of surface geometry [5–6], damage threshold [7–8], and so on. The laser-induced damage originates in photoionization of molecules caused by local electric fields generated at ultrafine structures in the substrates, such as indentations/protrusions in the surface or impurities in the interior. Recently, with advances made in the development of light sources, such as the trend towards lasers with higher power, shorter pulses, and shorter wavelengths, there has been a demand for the development of optical components having even higher damage resistance. Laser resistance is evaluated by an index called the laser-induced damage threshold (LIDT), which is measured by the 200-on-1 method according to ISO 11254-2 [9–10], for example. With this method, 200 locations on a sample surface are irradiated with single shots of pulsed laser light having different energy densities. The presence/absence of damage sites due to the irradiation is visually checked, and the minimum energy density at which damages are found is defined as the LIDT. However, the practical problem is that the performance of an optical component, such as transmittance, reflectance, etc., is degraded by continuous irradiation with laser light having an energy density below the LIDT. The cause of such degradation in performance, namely, laser-induced degradation of the optical component, is not fully understood. In addition, the resistance cannot be evaluated by tests based on ISO 11254-2, and therefore, a technique for the quantitative evaluation of such degradation would be useful. Furthermore, if the relation between laser-induced degradation and surface roughness is revealed, it may contribute to the development of a surface polishing technology for optical components with higher resistance against not only laser-induced damage but also degradation. Moreover, the clarification of the mechanism and knowledge about the details of the laser-induced degradation may provide an opportunity to develop novel laser processing techniques. Thus, our study about laser-induced degradation is a contribution to the progress in nanotechnology.

Therefore, we performed in situ a quantitative detection of laser-induced degradation in flat fused silica substrates, serving as the target material. The continuous irradiation with fs pulsed laser light has an energy density below the LIDT. We also analyzed the origin of the laser-induced degradation. Section 1 in Results and Discussion describes experiments in which we continuously irradiated substrates with fs pulsed laser light having a low energy density below the LIDT and detected the laser-induced degradation by measuring the scattered light. In addition, by simultaneously measuring the reflected-light spectrum during this process, we observed spectral changes as the laser-induced degradation proceeded. Section 2 in Results and Discussion describes the measurement of instantaneous reflectance changes using a pump–probe method carried out to analyze the details of these spectral changes. From an increase in photo-generated carriers and the power spectrum of their relaxation lifetime, we found that compositional changes in the molecular structure occurred as laser-induced degradation proceeded.

## Results and Discussion

### 1 Evaluation of laser-induced degradation of fused silica

#### 1.1 Surface roughness dependence of threshold fluence in laser-induced degradation

First, to quantitatively observe laser-induced degradation due to continuous irradiation with a laser light having an energy density below the LIDT, we conducted an experiment in which we continuously irradiated a silica substrate with fs laser light and measured the relative increase in scattered light from the substrate surface. As measurement samples, we used high-purity fused silica flat substrates in which the OH impurity content was 0.8 ppm or less. We used two samples: sample A, which was prepared by planarizing a substrate using standard chemical-mechanical polishing (CMP) [11], and sample B, which was prepared by performing optical near-field etching on a substrate prepared under the same conditions as sample A. The samples A and B had a minimum average surfaces roughnesses *R*_a_ of 0.20 nm and 0.13 nm, respectively [12]. Since we employed a continuous-wave laser with the wavelength of 532 nm and power of 2 W for optical near-field etching, the sample B did not have any laser-induced damage or degradation caused by this preparation. Optical near-field etching is a surface planarization technique for selectively removing only minute protrusions in the surface of a substrate, and can flatten the planer substrate polished by CMP. Thus, it is effective in reducing the drop in LIDT caused by electric field enhancement induced by the surface structure [5–6]. It has been reported that a dielectric multilayer mirror fabricated by using a silica substrate planarized by this technique exhibited an LIDT that was increased from 8.2 J/cm^2^ to 14 J/cm^2^ compared with the mirror before planarization processing, according to a 200-on-1 laser resistance test based on ISO 11254-2 [13].

**Figure 1:**
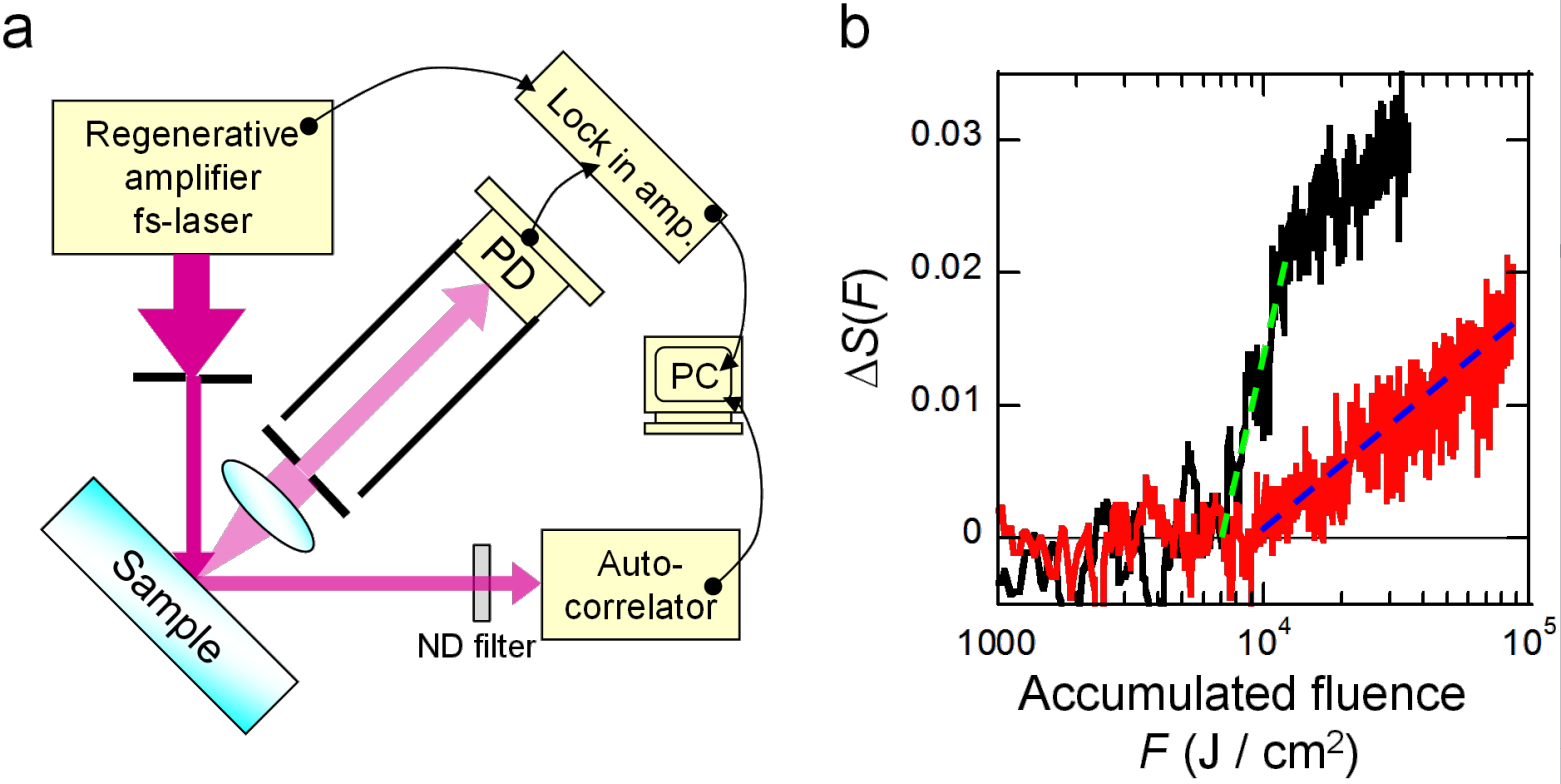
a) Schematic diagram of experimental set-up for measuring laser-induced degradation due to irradiation with energy density below the LIDT. b) Relative increase in scattered light intensity, Δ*S,* versus accumulated fluence, *F*, for samples A and B. Black and red solid lines show Δ*S*_A_ and Δ*S*_B_, respectively. The green and blue dotted lines show approximation curves *g*_A_(*F*) and *g*_B_(*F*) for regions where Δ*S*_A_ and Δ*S*_B_ rise.

Figure 1a shows the experimental set-up. For the light source, we used a regeneratively amplified fs Ti:sapphire laser (Coherent, Inc., Legend Elite F-1K) with a wavelength of 800 nm, a power of 1.0 W, a repetition frequency of 1 kHz, and a pulse width of 160 fs. The laser light was incident on the surface of the substrates at an incident angle of 45° and with an energy density of 6.0 mJ/cm^2^. This energy density is sufficiently lower than the LIDT of fused silica for the same wavelength and pulse width, namely, about 0.5 J/cm^2^ [14–15]. Scattered light from the samples was collected by a Si photodiode (Hamamatsu Photonics K.K., S3883) placed perpendicularly to the substrate, and lock-in detection was performed at the repetition frequency of the laser using a lock-in amplifier (Stanford Research Systems, Inc., SR830).

We evaluated the two silica substrates using the relative increase in the scattered light intensity *S,* Δ*S*(*F*) = (*S*(*F*) – *S*(0))/*S*(0), versus the accumulated fluence *F* (J/cm^2^) of the irradiated light. In the experiment, we continuously irradiated samples A and B with laser light having fluences up to *F* = 3.5 × 10^4^ J/cm^2^ and 8.8 × 10^4^ J/cm^2^, respectively, and after the experiment, we confirmed that there were no sites showing laser-induced damage in the surfaces of the samples by using an optical microscope. The results of measuring Δ*S*(*F*) for samples A and B, Δ*S*_A_ and Δ*S*_B_, are shown by the black and red solid lines in Figure 1b, respectively. We observed a tendency for the scattered light to increase with continuing irradiation, showing that this method succeeded in quantitatively measuring the laser-induced degradation. In addition, for both Δ*S*_A_ and Δ*S*_B_, the scattered light intensity increased logarithmically from specific fluences, showing that there was a threshold fluence at which laser-induced degradation started. To determine this threshold fluence, *F*_t_, the regions where Δ*S*_A_ and Δ*S*_B_ rose were fitted with an approximation *g*(*F*) = *P*log(*F*) − *Q*. The approximation curves are shown by the green and blue dotted lines in Figure 1b, where *g*_A_(*F*) was defined over the range 6.3 × 10^3^ J/cm^2^ < *F* < 1.2 × 10^4^ J/cm^2^ for Δ*S*_A_, and *g*_B_ (*F*) was defined over the range *F* > 9.2 × 10^3^ J/cm^2^ for Δ*S*_B_. The constants *P* and *Q* are as shown in Table 1. When *F*_t_ at which *g*_A_(*F*) = 0 and *g*_B_(*F*) = 0 is determined from these equations, for the samples A and B, we calculated *F*_tA_ = 6.3 × 10^3^ J/cm^2^ (1.1 × 10^6^ shots) and *F*_tB_ = 9.2 × 10^3^ J/cm^2^ (1.5 × 10^6^ shots), under the laser energy density of 6.0 mJ/cm^2^. This result shows that the degradation starting threshold for sample B is 1.4-times higher than that for sample A. As described above, sample B was a substrate prepared by selectively removing only the ultrafine surface structure from the substrate of sample A, and the LIDT of a mirror using a substrate identical to this was improved by a factor of 1.7 [13]. Since the degradation starting threshold *F*_t_ and the LIDT improvement factor are in good agreement, this result indicates the laser-induced degradation occurs with a partly similar mechanism of laser-induced damage, namely multiphoton ionization caused by local electric fields generated at indentations or protrusions. Also, the constants *P* which define the rate of increase of *g*_A_(*F*) and *g*_B_(*F*) are *P*_A_ = 0.062 and *P*_B_ = 0.016, differing by a factor of 3.9, which shows that laser-induced degradation originating from the surface structure proceeds more rapidly.

**Table 1:** Constants *P* and *Q* for approximation curves *g*_A_(*F*) and *g*_B_(*F*).

	*P*	*Q*

*g*_A_(*F*)	0.062	0.24
*g*_B_(*F*)	0.016	0.062

#### 1.2 In situ evaluation of spectral change in reflected light

To analyze the laser-induced degradation, we evaluated the reflected light spectrum at the same time as the scattered light intensity measurement. As shown in Figure 1a, reflected light was introduced from the sample into an autocorrelator with a spectrometer function (APE GmbH, pulseCheck). We used a fused silica substrate identical to sample A and set the energy density of incident light on the substrate to 17 mJ/cm^2^, which is below the LIDT. Spectral data was obtained at four fluences, namely, *F*_1_ = 5.1 × 10^3^ J/cm^2^, *F*_2_ = 1.9 × 10^5^ J/cm^2^, *F*_3_ = 5.9 × 10^5^ J/cm^2^, and *F*_4_ = 1.3 × 10^6^ J/cm^2^.

**Figure 2:**
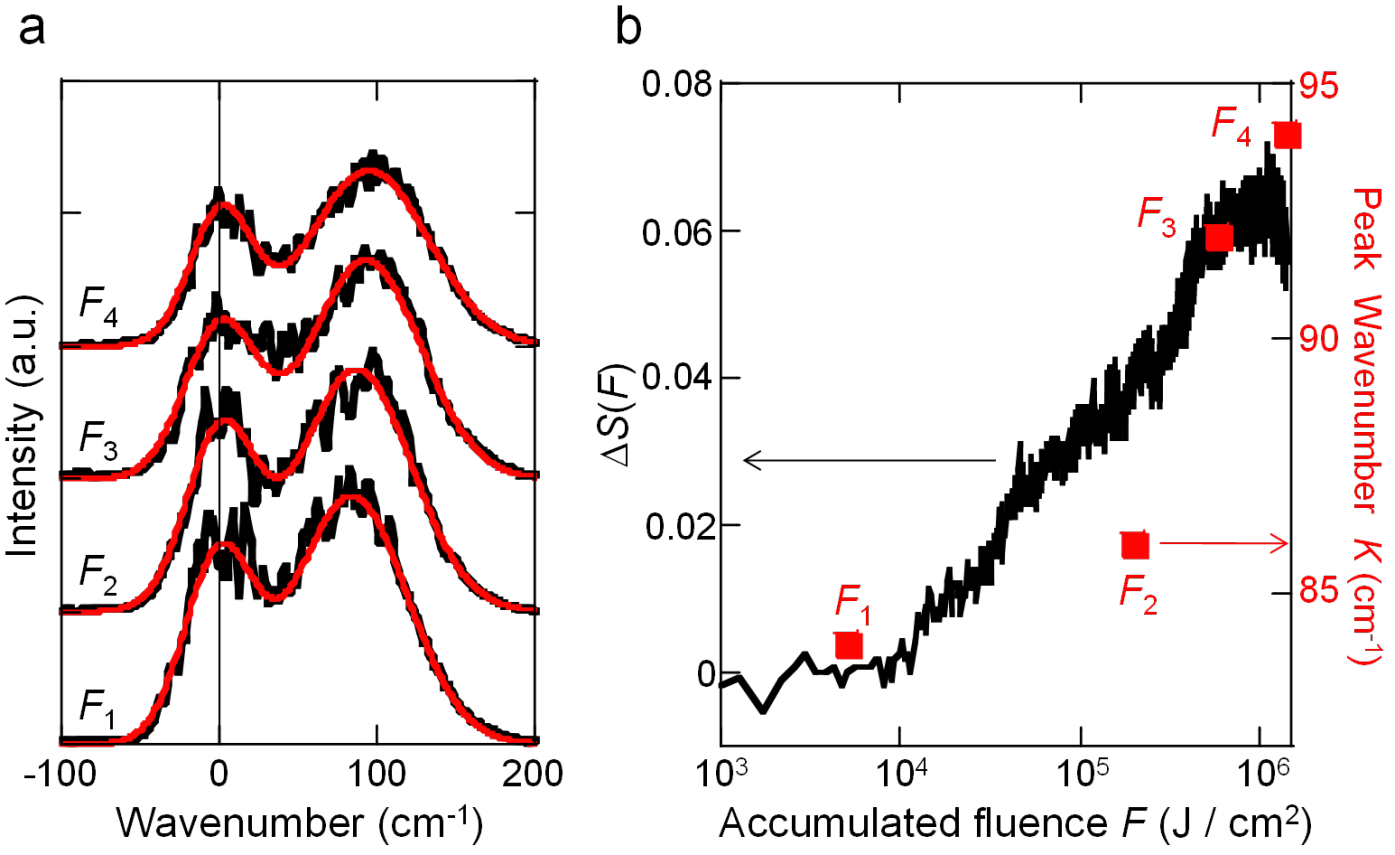
a) Wavenumber spectra of reflected light from silica substrate for accumulated fluences *F*_1_ to *F*_4_, where *F*_1_ = 5.1 × 10^3^ J/cm^2^, *F*_2_ = 1.9 × 10^5^ J/cm^2^, *F*_3_ = 5.9 × 10^5^ J/cm^2^, and *F*_4_ = 1.3 × 10^6^ J/cm^2^. The black and red solid lines indicate the measured values and approximation curves given by the sum of two Gaussian functions, respectively. The vertical axis shows arbitrary units and the baselines of *F*_1_ to *F*_4_ are essentially equivalent. b) Dependence of relative increase in scattered light intensity, Δ*S* (black line), and peak wavenumber in reflected light spectra *K* (red squares) on accumulated fluence, *F*.

The solid black line in Figure 2a shows the wavenumber spectra of the reflected light, centered on the central wavelength of the laser (800 nm). Under all conditions, in addition to the central frequency of the laser, we observed the 2nd peak on the long-wavelength side. When these wavenumber spectra were fitted with the sum of two Gaussian functions, as shown by the solid red lines in Figure 2a, the center wavenumbers on the 2nd peaks *K* were determined to be *K*_1_ = 84 cm^−1^, *K*_2_ = 86 cm^−1^, *K*_3_ = 92 cm^−1^, and *K*_4_ = 94 cm^−1^ for the fluences *F*_1_ to *F*_4_, respectively. The relative increase in the scattered light intensity, Δ*S*, and *K* versus the irradiated fluence, *F*, are plotted in the black solid line and the red squares in Figure 2b, respectively. The monotonically increasing trends with respect to *F* agreed well in both Δ*S* and *K*, showing that the 2nd peak of the spectrum shifted to lower energy side as the degradation proceeded. We estimated the origin of the 2nd peaks were Raman signals of degraded silica and this result represented the compositional changes in the molecular proceeds with the laser-induced degradation proceeded.

### 2 Analysis of degradation via pump–probe method

#### 2.1 Time-resolved measurement of reflectance

**Figure 3:**
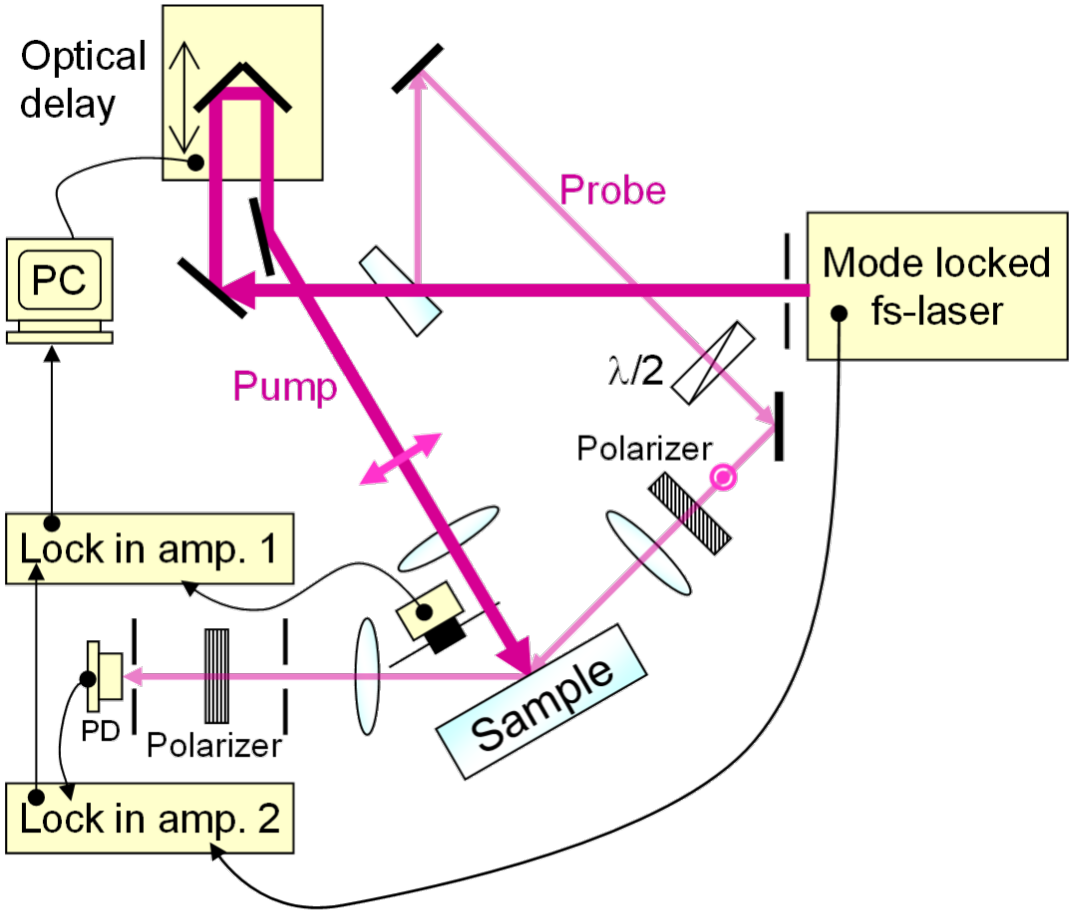
Schematic diagram of pump–probe experimental set-up.

To analyze in detail the spectral changes occurring with laser-induced degradation due to continuous irradiation with laser light having an energy density below the LIDT, as confirmed in the previous section, we conducted an experiment to evaluate the time-resolved changes in reflectance via a pump–probe method. The experimental setup is shown in Figure 3. The laser used was a mode-locked Ti:sapphire laser (Coherent, Inc., Mira Optima 900-D) with a wavelength of 750 nm, a pulse width of 90 fs, and a repetition frequency of 80 MHz. Using a beamsplitter, the laser light was split with an intensity ratio of reflected light to transmitted light of 1:19. As pump light, the transmitted light was made perpendicularly incident on the sample surface via an optical delay and a chopper with a frequency of 2 kHz. P-polarized probe light, with a polarization orthogonal to that of the pump light, was incident on the substrate at an angle of incidence of about 70°, and the reflected probe light was received by a Si photodiode (Hamamatsu Photonics K.K., S3883). The received signal was detected with double lock-in detection using two lock-in amplifiers (Stanford Research Systems, Inc., SR844 and SR830) based on the repetition frequency of the laser pulses and the frequency of the chopper. The laser light used as the pump light in this experiment had an energy density of 1.6 μJ/cm^2^, and no increase in light scattering was observed even when the silica substrate was continuously irradiated with this laser light with a accumulated fluence exceeding 1.0 × 10^7^ J/cm^2^, thus confirming that the progression of laser-induced degradation could be ignored in this pump–probe experiment. We performed measurements on three samples, which were all high-purity fused silica substrates polished by CMP, identical to sample A: Sample C, on which no laser was irradiated; Sample D, which showed laser-induced degradation by being irradiated with the regeneratively amplified fs laser with a wavelength of 800 nm, an energy density of 17 mJ/cm^2^, a repetition frequency of 1kHz and a pulse width of 160 fs to an accumulated fluence of 3.5 × 10^4^ J/cm^2^, namely 2.1 × 10^7^ shots; and Sample E, in which laser-induced damage visually recognizable as damage sites was caused by 1000 shots of irradiation with an energy density of 100 mJ/cm^2^ using the same fs laser.

**Figure 4:**
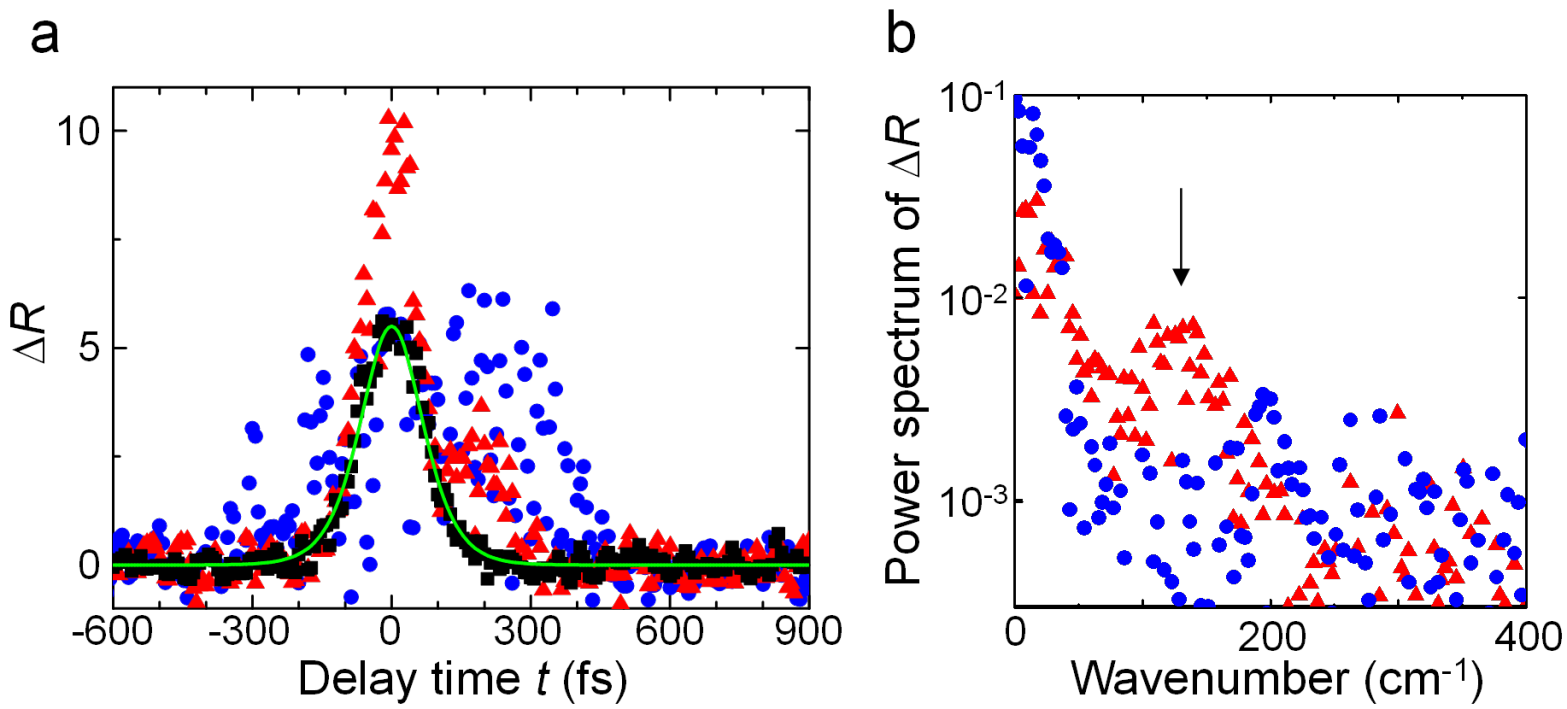
a) Instantaneous relative increase in reflected light intensity, Δ*R*, measured by pump–probe method. Black squares, blue circles, and red triangles indicate measurement results for samples C, D, and E, respectively. The solid green line indicates the approximation curve Δ*R*_fit_ = 5.5 sech^2^(*t*/(9 × 10^−14^)) for Δ*R*_C_. b) Power spectrum obtained by subtracting the Δ*R*_fit_ component from Δ*R*_D_, and Δ*R*_E_. The blue circles and red triangles indicate the analysis results for Δ*R*_D_, and Δ*R*_E_, respectively. The arrow indicates 128 cm^−1^.

In the evaluation, we used a relative change Δ*R* = (*R* – *R*_0_)/R_0_, where *R*_0_ is the reflected light intensity in the case where only the probe light was radiated, and *R* is the reflected light intensity obtained in the pump–probe experiment. The measurement results Δ*R*_C_, Δ*R*_D_, and Δ*R*_E_ for samples C, D, and E are plotted in Figure 4a as black squares, blue circles, and red triangles, respectively. The horizontal axis of the graph is the time delay, *t*, of the probe light relative to the pump light, where the peak of Δ*R*_C_ is taken as *t* = 0. Δ*R*_C_ is well-approximated by Δ*R*_fit_ = 5.5 sech^2^(*t*/(9 × 10^−14^)), which is shown by the green solid line in Figure 4a, and nonlinear polarization reflecting the pulse width of the laser was observed. In contrast, Δ*R*_D_, and Δ*R*_E_ showed temporal broadenings of about several 100 fs. Because these decay times are close to the photogenerated carrier lifetime of silica 150 fs [16–17], it is assumed the temporal changes in the reflectance were originated from the photogenerated carrier in samples D and E. In other words, in Samples D and E, we concluded that the generation probability of photogenerated carriers was higher than in Sample C because electric field enhancement occurred due to laser-induced degradation and laser-induced damage.

#### 2.2 Spectrum analysis

To analyze this in more detail, we obtained the power spectrum by subtracting the nonlinear polarization component represented by Δ*R*_fit_ from Δ*R*_D_, and Δ*R*_E_. The results are shown by the blue circles and red triangles in Figure 4b, respectively. For sample D, a signal having a weak peak close to 195 cm^−1^ was obtained, whereas for sample E, a broad peak around 128 cm^−1^ was found, thus giving different signals. The peak at 128 cm^−1^ was consistent with the Raman signal of α-quartz, which is formed of six-membered rings of Si and O [18]. It has been reported that the Raman signal from silica with a specific molecular structure is increased by irradiation with fs laser light having an energy density above the LIDT [19], and sample E is also thought to be mainly formed of six-membered rings in a similar fashion. Although there is no molecular structure of silica that corresponds to the peak for sample D, the result indicates that compositional changes occur due to laser-induced degradation. As we found in section 1.1, the laser-induced degradation originates in multiphoton ionization. The laser-induced damage by fs laser occurs by multiphoton ionization and consequent avalanche ionization [3–4]. Since there are no breakdowns, it is estimated that avalanche ionization does not occur in the laser-induced degradation due to its low energy density. While the carriers generated by just multiphoton ionization are not enough to cause breakdown, they change amorphous silica to a more stable molecular structure. As also indicated in the results in section 1.2, the spectrum shifted, namely the compositional change carried on as laser-induced degradation proceeds. It provides nanoscale nonuniformity of the refractive index which increases scattering of light, decreases transmittance, and accelerates the progress of the laser-induced degradation.

## Conclusion

In this paper, we focused on the degradation in performance of optical components due to continuous irradiation with laser light having an energy density below the LIDT, i.e., laser-induced degradation. We examined the degradation of fused silica substrates in response to fs laser irradiation and performed an in situ observation of the laser-induced degradation, as well as an analysis of the underlying mechanism. By monitoring the scattered light intensity versus the accumulated irradiation fluence, we succeeded in quantitatively detecting the laser-induced degradation. We confirmed that the total irradiation threshold at which laser-induced degradation starts changed depending on the size of the ultrafine structure in the surface. In addition, we also found that the spectrum of reflected light shifted as the laser-induced degradation proceeded. By analyzing the details of this behavior with a pump–probe method, we observed an increase in photogenerated carriers, and from the power spectrum, we obtained results indicating that the molecular structure of the silica undergoes compositional changes due to the laser-induced degradation.

From the findings we obtained in this study, it can be concluded that, to increase the resistance of a substrate to laser-induced degradation, it is effective to improve the substrate's flatness, similarly to the case of the LIDT. In addition, because it is possible to estimate the molecular structure formed by the laser-induced degradation from the spectral information, there is a possibility of achieving more effective measures against laser-induced degradation. The measurement method employed here for the fs laser light and the flat fused silica substrates can also be applied to other types of laser and optical components and is expected to contribute to the development of optical components having high resistance to laser-induced degradation in the future.
